# Developing an Indonesian fertility preservation questionnaire for health care providers treating patients with cancer: A preliminary pilot study

**DOI:** 10.12688/f1000research.15948.2

**Published:** 2019-05-10

**Authors:** Achmad Kemal Harzif, Raymond Surya, Mila Maidarti, Ana Mariana, Bara Tracy Lovita, Budi Wiweko

**Affiliations:** 1 Division of Immuno-Endocrinology and Fertility, Department of Obstetrics and Gynecology, Faculty of Medicine, Universitas Indonesia, Dr. Cipto Mangunkusumo Hospital, Jakarta, DKI Jakarta Province, Indonesia; 2 Department of Obstetrics and Gynecology, Faculty of Medicine, Universitas Indonesia, Dr. Cipto Mangunkusumo Hospital, Jakarta, DKI Jakarta Province, Indonesia; 3 Indonesian Reproductive Medicine Research and Training Center (INA-REPROMED), Faculty of Medicine, Universitas Indonesia, Dr. Cipto Mangunkusumo Hospital, Jakarta, DKI Jakarta Province, Indonesia

**Keywords:** Knowledge, Attitude, Practice, Indonesian Questionnaire

## Abstract

**Background: **Early detection and advanced treatment increases the five-year survival rate of patients with cancer. However, long-term cancer therapy, such as chemotherapy and radiotherapy, can have negative effects, such as infertility. This study aimed to develop a standardized Indonesian questionnaire, which would be used to assess the quality of health care providers’ knowledge, attitude, and practice regarding fertility preservation in patients with cancer.

**Methods: **A pilot study was performed in January and February 2018 at Dr. Cipto Mangunkusumo Hospital, Jakarta, Indonesia. An existing questionnaire was translated from English to Indonesian using forward translation, back translation, expert panel, pretesting, and cognitive interviewing. Ten subspecialists in the following departments made up an expert panel, who were involved in pretesting and cognitive interviewing: pediatric hematology-oncology, hematology-oncology/internal medicine, gynecologic oncology, gynecologic immune-endocrinology, radiology-oncology, and surgical oncology.

**Results: **The questionnaire was successfully translated. The ten respondents stated that the maximum age for women’s fertility preservation is 40 years of age (60%), 45 years of age (30%), or had no maximum age (10%). Additionally, the respondents stated that the maximum age for men’s fertility preservation is 40 years of age (30%), 50 years of age (20%), or had no maximum age (50%). The respondents’ knowledge stated that > 50% of them were aware but do not know enough about fertility preservation. The respondents stated that more than 50% of them give feedback agreeing to fertility preservation, and they always give advice about fertility preservation to their patients.

**Conclusion:** The translation of the questionnaire followed translation steps from the World Health Organization and was adjusted based on the expert panel’s comments concerning fertility preservation. This validated questionnaire tool in Indonesian can be used for research purposes and clinical evaluation of fertility preservation among health care providers in Indonesia.

## Introduction

Based on data from the US Cancer Statistics Working Group, the five most common cancers in 2014 were breast, prostate, lung and respiratory tract, colon and rectum, and uterine and ovary ^1^. The *Riset Kesehatan Dasar 2013* showed that the prevalence of cancer in Indonesia was 0.14% (347,782 people), with cervical (0.08%; 98,692 people) and breast (0.05%; 61,682 people) cancers ranked highest ^2^. In Indonesia, more than 135,000 people below 45 years of age are diagnosed with cancer annually ^2^.

Early detection and advanced treatments increase the five-year survival rate of patients with cancer. However, long-term cancer therapy, such as chemotherapy and radiotherapy, can have negative psychologic, economic, social, sexual, and biologic effects ^3, 4^. In 2014, the Guidelines from National Comprehensive Cancer Network stated that fertility preservation is an essential component when treating young and adolescent patients with cancer ^5, 6^. In fact, few patients are offered treatment choices based on fertility preservation due to lack of knowledge on optimal time, methods, and counselling approaches ^7^. The American Academy of Pediatrics, American Society of Clinical Oncology, and American Society of Reproductive Medicine suggest discussing potential complications and choice of fertility preservation as early as possible ^8, 9^.

Because there are no fertility preservation questionnaires available in Indonesia, this study aimed to develop a standardized Indonesian questionnaire that can be used to assess quality of health care providers’ knowledge, attitude, and practice regarding fertility preservation in patients with cancer.

## Methods

### Ethical statement

This study was approved by The Ethics Committee of Faculty of Medicine, University of Indonesia under number 926/UN2.F1/ETIK/2017. Written informed consent was obtained from all participants prior to participation.

### Questionnaire translation

This study was performed in January and February 2018 at Dr. Cipto Mangunkusumo Hospital, Jakarta, Indonesia. An existing questionnaire, “Fertility preservation in cancer survivors: A national survey of oncologists’ current knowledge, practice, and attitudes,” published in English in 2013 ^10^ was translated to Indonesian by two independently certified medical translators whose first language is Indonesian, with permission from the publisher.

To check the accuracy of the translation, the Indonesian questionnaire was back translated to English by another medical translator (RS). Misunderstandings or unclear word choices in the initial translations were resolved by an author (RS) as appropriate to the aim of this study. After the translation had been completed, an expert (AKH) familiar with the construct of interest and methodology reviewed all versions of translations and determined whether the translation had achieved semantic, idiomatic, experiential, and conceptual equivalence.

The final translation of the Indonesian pilot questionnaire ( Supplementary File 1) was given to specialists and subspecialists who completed the questionnaire and were interviewed verbally to ensure clarity of answers ^11^. These interviews took place in Dr. Cipto Mangunkusumo Hospital. Specialist and subspecialists were chosen randomly from all staff at Dr. Cipto Mangunkusumo Hospital who fit the inclusion criteria (see below). Researchers contacted these experts directly, providing an explanation of the study and gaining informed consent from the expert to get involved in this study. Interviews were not recorded.

### Questionnaire validation

Ten subspecialists or specialists in the following departments who directly treat patients with cancer were recruited to take part in the study: pediatric hematology-oncology, hematology-oncology/internal medicine, gynecologic oncology, gynecologic immune-endocrinology, radiology-oncology, and surgical oncology. The subspecialist or specialist in Dr. Cipto Mangunkusumo Hospital were randomly chosen and recruited to this study based on their experience for at least 5 years. They were considered as an expert panel. Inclusion criteria for this study were (a) subspecialists or specialists aged 30–45 years; and (b) subspecialists or specialists who have studied in their field for at least 5 years; and exclusion criteria for this study were (a) respondents who not willing to be a participant in this study; and (b) incomplete filling of the informed consent.

Due to the qualitative nature of this study, only face validity and construct validity were assessed. Face validity was used to determine whether the instrument was understandable and relevant to the targeted population. Construct validity was used to determine the reason and consequence describing the real condition. Content validity was not assessed because the purpose of this questionnaire was not to determine good/bad knowledge and positive/negative attitude about practice.

### Data analysis

The data gathered from completed questionnaires were distributed by frequency and percentage. The analysis used SPSS Statistics for Windows, version 23.0.

## Results

In this preliminary pilot study, ten respondents from Dr Cipto Mangunkusumo Hospital (8 males) participated in this study from the following specialties: pediatric hematology-oncology (n=2), hematology-oncology/internal medicine (n=1), gynecologic oncology (n=2), gynecologic immune-endocrinology (n=2), radiology-oncology (n=1), and surgical oncology (n=2).

### Responses to the completed questionnaires

In total, 60% (6/10), 30% (3/10), and 10% (1/10) of respondents stated that the maximum age for women’s fertility preservation is 40 years of age, 45 years of age, or had no maximum age, respectively. A total of 30% (3/10), 20% (2/10), and 50% (5/10) of respondents stated the maximum age for men’s fertility preservation is 40 years of age, 50 years of age, or had no maximum age, respectively.

Table 1 describes the frequency that health care providers encounter patients who have used/are using fertility preservation options. Table 2 describes the familiarity of health care providers about methods of fertility preservation.

**Table 1.** Frequency of health care providers encountering patients who have used/are using fertility preservation options.

	Never	Rarely	Usually	Always
Ovarian tissue cryopreservation	8/10	2/10	0	0
Oocyte cryopreservation	6/10	3/10	1/10	0
*In vitro* fertilization with embry o cryopreservation	6/10	0	1/10	3/10
Sperm cryopreservation	7/10	0	0	3/10
Testicular tissue cryopreservation	8/10	2/10	0	0
Pre-treatment cancer using GnRH agonist	5/10	2/10	1/10	2/10

**Table 2.** Health care providers’ knowledge of fertility preservation options.

	Not at all knowledgeable	Aware of but do not know well	Knowledgeable	Very knowledgeable
Ovarian tissue cryopreservation	0	6/10	4/10	0
Oocyte cryopreservation	0	5/10	3/10	2/10
*In vitro* fertilization with embryo cryopreservation	0	6/10	1/10	3/10
Sperm cryopreservation	0	5/10	3/10	2/10
Testicular tissue cryopreservation	2/10	6/10	2/10	0
Pre-treatment cancer using GnRH agonist	0	5/10	5/10	0

Table 3 shows the health care providers’ practice of giving advice about fertility preservation, and Table 4 shows their attitudes towards fertility preservation. Table 5 shows the factors influencing health care providers when initiating a discussion about fertility preservation.

**Table 3.** Health care providers’ practice of giving advice about fertility preservation.

	Never	Rarely	Usually	Always
I consider how essential fertility is in the future	0	3/10	2/10	5/10
When I plan the patient’s treatment regimen, I take into account their desire for future fertility	1/10	3/10	0	6/10
I discuss the impact of a patient’s condition and/or treatment might have on their future fertility	0	0	2/10	8/10
I provide my patients with written information about fertility preservation	1/10	3/10	1/10	5/10
I consult a fertility specialist or reproductive endocrinologist with questions about potential fertility issues of my patients	3/10	4/10	1/10	2/10
I refer patients who have questions about fertility to a fertility specialist or reproductive endocrinologist	5/10	3/10	0	2/10

**Table 4.** Health care providers’ attitudes towards fertility preservation.

	Strongly disagree	Disagree	Neither agree or disagree	Agree	Strongly agree
Fertility preservation is a high priority to discuss with newly diagnosed cancer patients	0	0	0	7/10	3/10
Treating the primary cancer is more important than fertility preservation	0	2/10	2/10	4/10	2/10
The success rates of fertility preservation are not as yet good enough to make it a viable option	0	3/10	6/10	1/10	0
I feel comfortable discussing fertility preservation with my patients	0	1/10	1/10	6/10	2/10
I am willing to provide a less effective cancer treatment regimen in order to attempt to preserve a patients’ fertility	0	6/10	0	2/10	2/10

**Table 5.** Factors that health care providers consider when deciding to initiate a discussion about fertility preservation.

Factors	Not at all	To some extent	To a large extent
Poor success rates of fertility preservation options	1/10	5/10	4/10
Lack of fertility services in the area	2/10	3/10	5/10
Constraints on my time	4/10	5/10	1/10
My limited knowledge of fertility preservation options	1/10	8/10	1/10
Burden to patients	1/10	6/10	3/10
Someone else within my practice discusses fertility preservation with my patients	4/10	2/10	4/10
The patient …			
… is too ill to delay treatment to pursue fertility preservation	0	7/10	3/10
… cannot afford fertility preservation	3/10	6/10	1/10
… has a hormonally-sensitive malignancy	0	7/10	3/10
… does not want to discuss fertility preservation	2/10	7/10	1/10
… has a poor prognosis	0	5/10	5/10
... is single	0	6/10	4/10
… is lesbian or gay	8/10	2/10	0
… already has a child or children	1/10	7/10	2/10

### Feedback from the participants about the questionnaire

All comments have been translated from Indonesian.

The following are comments made during the verbal interview about the format and definitions used in the translated questionnaire:

*“The questionnaire should contain [an] explanation of each fertility preservation.”* (hematology-oncologic internal medicine.)
*“The identity [of a patient requiring fertility preservation] is only initial. [The] number of cases on Q2 should be made [into categories] and focused to the last year. [The] format [of the] questionnaire should be adjusted to [be easier for] the readers.”* (hematology-oncologic pediatrician.)
*“The questionnaire is too difficult. It should contain the explanation of what the definition [is for] each fertility preservation.”* (hematology-oncologic pediatrician.)
*“The length of clinical practice in the oncology field should be included to the questionnaire.”* (oncology gynecologic.)
*“On identity column, it should add the last major educational background and radio-oncology should be included. Number of cases taking care by health care providers should be shown [by] percentage.”* (radio-oncology.)

The following are comments made during the verbal interview about the use of cultural background used in the translated questionnaire:

*“Cultural background around social, racial, and religion should be omitted.”* (hematology-oncologic internal medicine.)
*“On cultural background [questions], [due to the] culture [in] Indonesia [concerning] gay or lesbian, or towards social [status], race, and religion should be omitted to minimalize the possibility of conflict.”* (immune endocrinology gynecologic.)

All raw data included the medical background of respondents, the health care providers’ knowledge, attitude, and practice regarding fertility preservation and also factors that health care providers consider when deciding to initiate a discussion about fertility preservation Click here for additional data file. Copyright: © 2019 Harzif AK et al. 2019 ${data-license-text}

## Discussion

This study includes the pilot results of the measures of success of a translation of an existing questionnaire to Indonesian to determine knowledge, attitude, and practice of providers regarding fertility preservation in patients with cancer. The results of this study are not applicable to all health care providers because the questionnaire is very specific for clinicians treating patients with cancer.

The World Health Organization proposes various steps to achieve different language versions of English instruments that are equivalent in each target country/culture: forward translation, expert panel, back translation, pretesting and cognitive interviewing, leading to a final version ^12^. In this study, the expert panel, made up of ten subspecialists and specialists, provided feedback after the back translation via pretesting and cognitive interviewing. After this pilot study, we would like to distribute this questionnaire to health care providers treating patients with cancer.

Based on respondents’ feedback, we conclude that, in Indonesia, fertility preservation still is not common and familiar among practitioners taking care of patients with cancer. In total, 50% of respondents were aware of but not experts in fertility preservation. Additionally, 50% of respondents never had patients who had used or were using fertility preservation; however, respondents were subspecialist oncologists or clinicians directly taking care of patients with cancer. This may be because there is still no availability of fertility preservation in Indonesia. In Hong Kong, 45.6% of clinicians were familiar with fertility preservation ^13^.

Our study also shows that most respondents had discussed the impact of treatment to future fertility with patients. In total, 30% of respondents had referred patients to a fertility specialist. A similar study in Lebanon found that 90% of clinical practitioners and 94% of oncologists agreed to discuss fertility preservation with patients before cancer treatment ^14^. Clinicians in Hong Kong did not refer patients to fertility specialists due to lack of available time before treatment, considerable risk of recurrence, poor prognosis, financial constraints, cancer treatment as top priority at the time, and lack of awareness of such service ^13^.

A comment said that difficulty of this questionnaire was about the explanation for each definition of fertility preservation. The providers treating patients with cancer were not familiar for options of fertility preservation. Harzif, *et al.* ^15^ proved that most obstetricians and gynaecologists knew about fertility preservation (86.3%); however, they were not familiar enough for each option. The knowledge of sperm, oocyte, embryo, ovarian tissue, testicular tissue preservation, and pre-treatment with GnRH agonist was less than 50%. Moreover, other specialists treating cancer patients might not be familiar enough for each option of preservation. Therefore, this Indonesian questionnaire should include the definition for each method of preservation. Apart from that, issue about gay, lesbian, race, and religion was still taboo in Indonesia.

## Limitations

As shown by the quotes in the Results section, limitations of the questionnaire were that cultural background factors influenced health care providers’ decisions to initiate discussions about fertility preservation. The questions about this issue can result contradiction between pro and contra of this issue. That is why respondents suggested to omit it for Indonesian questionnaire. For example, British respondents stated that their decision to discuss fertility preservation was influenced by poor prognosis (88%) and whether the patient already had children (45%) ^10^.

## Conclusion

Based on data obtained in this preliminary pilot study, we translated the English questionnaire to Indonesian and revised it following processes adopted from World Health Organization and adjusted through expert respondents’ comment. Supplementary File 1 contains the Indonesian version of the questionnaire about the quality of health care providers’ knowledge, attitude, and practice regarding fertility preservation in patients with cancer.

By having this validated tool questionnaire in Indonesian, it can be used for both research purposes and clinical evaluation of fertility preservation among health care providers in Indonesia. 

## Data availability

F1000Research: Dataset 1. All raw data included the medical background of respondents, the health care providers’ knowledge, attitude, and practice regarding fertility preservation and also factors that health care providers consider when deciding to initiate a discussion about fertility preservation, https://doi.org/10.5256/f1000research.15948.d227878 ^16^

## Supplementary material

Supplementary File 1: Questionnaire and informed consent form in Bahasa Indonesia.
