# Supplementary material for: Developing an Indonesian fertility preservation questionnaire for health care providers treating patients with cancer: A preliminary pilot study
Source: F1000Res. 2019 May 10;7:1890. Originally published 2018 Dec 4. [Version 2] doi: 10.12688/f1000research.15948.2 (PMC6544131; doi:10.12688/f1000research.15948.2)
Supplement: Supplementary file 2 [file f1000research-7-21101-s0001.tgz › 961cd2f9-f202-4183-997d-e90576470d2d_Questionnaire_and_Form_Informed_Consent_in_Bahasa_Indonesia_(Supplementary_File_1).docx]

| **FORMULIR PERSETUJUAN MENGIKUTI PENELITIAN**  **(FORMULIR INFORMED CONSENT)** | | | |
| --- | --- | --- | --- |
| Peneliti Utama | | : dr. Achmad Kemal Harzif, Sp.OG(K) | |
| Peneliti Anggota | | : dr. Raymond Surya | |
| Pemberi informasi | | : | |
| Penerima informasi  Nama Subyek  Tanggal Lahir (Umur)  Jenis Kelamin  Alamat  No. Telp (HP) | | :  :  :  :  :  : | |
|  | **JENIS INFORMASI** | **ISI INFORMASI** | **TANDAI JIKA SUDAH DIBACA** |
| 1. | Judul Penelitian | Profil Pengetahuan, Sikap, Perilaku Dokter yang Melayani Pasien Kanker Mengenai Preservasi Fungsi Reproduksi |  |
| 2 | Latar Belakang | Deteksi dan penatalaksanaan yang baik di bidang kanker membuat angka harapan hidup pada pasien kanker meningkat. Salah satu perhatian pada pasien kanker ialah fungsi reproduksi yang berkaitan dengan kualitas hidup. Walaupun preservasi fungsi reproduksi sudah dianggap sebagai standar perawatan pasien kanker, namun masih banyak pasien belum ditawarkan pilihan ini. Hal ini dapat disebabkan oleh kurangnya pengetahuan tentang waktu yang optimal, cara tindakan, serta pendekatan konseling preservasi fungsi reproduksi. |  |
| 3. | Tujuan Penelitian | Mengetahui gambaran profil pengetahuan, sikap, dan perilaku dokter yang melayani pasien kanker mengenai preservasi fungsi reproduksi |  |
| 4. | Cara dan Prosedur Penelitian | Yth dokter, pada kesempatan ini saya meminta dokter untuk berpartisipasi dalam penelitian ini. Jika dokter bersedia, maka dokter diminta untuk mengisi lembar kesediaan ikut serta. Peneliti akan memberikan penjelasan mengenai penelitian ini terhadap dokter. Dokter diharapkan dapat mengisi kuesioner penelitian ini dengan jujur dan lengkap demi kualitas hasil penelitian ini.  Tidak ada resiko dan efek samping yang berarti dari pengisian kuesioner ini bagi dokter.  Selain itu, keikutsertaan dokter dalam penelitian bersifat sukarela dan tidak ada paksaan. Dokter berhak menolak apakah akan berpartisipasi atau tidak. |  |
| 5. | Jumlah subjek | 10 dokter spesialis atau dokter subspesialis |  |
| 6. | Manfaat Penelitian termasuk manfaat bagi subyek penelitian | Memberikan data untuk dapat melakukan evaluasi terhadap pelayanan kesehatan di bidang preservasi fungsi reproduksi.  Mengembangkan petunjuk bagi dokter yang melayani pada preservasi fungsi reproduksi  Memberikan data dasar sebagai bahan untuk melakukan evaluasi pendidikan dokter spesialis di Indonesia  Sebagai dasar untuk pengembangan pengetahuan obstetri dan ginekologi khususnya dalam bidang preservasi fungsi reproduksi |  |
| 7. | Risiko dan Efek samping dalam penelitian | Tidak ada |  |
| 8. | Ketidaknyamanan subyek penelitian | Kelelahan dalam pengisian kuesioner |  |
| 9. | Kompensasi bila terjadi efek samping | Tidak ada |  |
| 10. | Alternatif penanganan (bila ada) | Tidak ada |  |
| 11. | Penjagaan kerahasiaan data | Data yang telah diambil oleh peneliti langsung disimpan di satu folder. Hanya peneliti yang dapat mengakses data tersebut. |  |
| 12. | Biaya yang ditanggung oleh subyek | Tidak ada |  |
| 13. | Insentif bagi subyek | Tidak ada |  |
| 14. | Nama dan alamat peneliti serta nomor telepon yang dapat dihubungi | dr. Achmad Kemal Harzif, Sp.OG/  dr. Raymond Surya  Alamat: Departemen OG RSCM/ FKUI  HP: 081320000288 |  |

Setelah mendengarkan penjelasan pada halaman 1 dan 2 mengenai penelitian yang akan dilakukan oleh **dr. Achmad Kemal Harzif, Sp.OG(K)** dengan judul: **Profil Pengetahuan, Sikap, Perilaku Dokter yang Melayani Pasien Kanker Mengenai Preservasi Fungsi Reproduksi**, informasi tersebut telah saya pahami dengan baik.

Dengan menandatangani formulir ini, saya menyetujui untuk diikutsertakan dalam penelitian di atas dengan suka rela tanpa paksaan dari pihak manapun. Apabila suatu waktu saya merasa dirugikan dalam bentuk apapun, saya berhak membatalkan persetujuan ini.

_________________________ ______________________

………………………………. ……………………………...

(Pemberi informasi) (Subjek)

**KUESIONER PENGETAHUAN, SIKAP, DAN PERILAKU DOKTER YANG MELAYANI PASIEN KANKER MENGENAI PRESERVASI FUNGSI REPRODUKSI**

* Kuesioner dari Oxford Brookes University yang dimodifikasi di Indonesia

No. Kuesioner:

|  | |  |  |  |  |  |
| --- | --- | --- | --- | --- | --- | --- |
| Tanggal Pengisian | | : | | | | |
| Lokasi Pengisian | | : | | | | |



Tanda )* : Coret yang tidak perlu

**IDENTITAS**

| 1. | Inisial Nama | : |  |
| --- | --- | --- | --- |
| 2. | Jenis kelamin | : | Laki-laki / Perempuan |
| 3. | Alamat | : |  |
| 4. | No Telepon | : |  |
| 5. | Tempat/Tanggal lahir | : |  |
| 6. | Universitas pendidikan terakhir | : |  |
| 7. | Tahun lulus pendidikan terakhir | : |  |
| 8. | Lama praktik di bidang onkologi | : | ________ tahun |
| 9. | Agama | : |  |
| 10. | Suku | : |  |

**KUESIONER**

**Q1 Apakah latar belakang Anda?**

- Dokter umum
- Dokter spesialis. Apakah spesialisasi Anda?
  - Penyakit dalam
  - Penyakit dalam subspesialisasi hematologi onkologi medis
  - Anak
  - Anak subspesialisasi hematologi onkologi
  - Obstetri dan ginekologi
  - Obstetri dan ginekologi subspesialisasi onkologi dan ginekologi
  - Obstetri dan ginekologi subspesialisasi imunoendokrinologi reproduksi
  - Bedah umum
  - Bedah onkologi
  - Onkologi radiasi
  - Lain-lain: ______________

**Q1a Subspesialisasi kanker**

Jika Anda dokter subspesialis, apakah Anda memiliki spesialisasi di bidang kanker tertentu?

- Payudara
- Ginekologi
- Urologi
- Gastrointestinal
- Paru-paru
- Kepala-leher
- Hematologi
- CNS
- Anak
- Lain lain, sebutkan:
- Tidak ada spesialisasi

**Q2 Jumlah kasus pasien**

Berapa banyak perkiraan jumlah pasien yang Anda rawat rentang umur dibawah ini dalam **SATU tahun terakhir**:

0-17 tahun : kasus

18-45 tahun : kasus

>46 tahun : kasus

**Q3 Distribusi jenis kelamin kasus**

Dalam **1 bulan**, berapa persen perkiraan pasien onkologi Anda **berusia 18 hingga 45 tahun** (dibandingkan dengan seluruh rentang usia pasien onkologi yang ditangani)

| Laki-laki | ____ % |
| --- | --- |
| Perempuan | ____ % |

**Q4 Frekuensi kontak dengan pilihan-pilihan preservasi kesuburan**

Seberapa sering Anda bertemu pasien yang telah/sedang menggunakan salah satu dari pilihan-pilihan preservasi kesuburan berikut ini?

| **Mohon centang satu boks untuk masing-masing baris** | **Tidak pernah** | **Jarang** | **Kadang-kadang** | **Sering** |
| --- | --- | --- | --- | --- |
| Preservasi beku jaringan ovarium (*ovarian tissue cryopreservation*) |  |  |  |  |
| Preservasi beku oosit (*oocyte cryopreservation*) |  |  |  |  |
| Fertilisasi in vitro dengan preservasi beku embrio (*in vitro fertilization with embryo cryopreservation*) |  |  |  |  |
| Preservasi beku sperma (*sperm cryopreservation*) |  |  |  |  |
| Preservasi beku jaringan testis (*testicular tissue cryopreservation*) |  |  |  |  |
| Penanganan sebelum tatalaksana kanker (*pre-treatment*) dengan agonis GNRH (seperti suntikan depot leuprolide) |  |  |  |  |

**Keterangan:**

* Preservasi beku jaringan ovarium ialah mengambil bagian luar (korteks) ovarium yang mengandung banyak telur imatur dan kemudian dilakukan pembekuan. Keuntungan ialah dapat membekukan jaringan yang berisi telur imatur berjumlah ratusan hingga ribuan untuk penggunaan yang akan datang.

* Preservasi beku oosit ialah pembekuan ovum yang diambil melalui stimulasi ovarium dan sel telur tidak difertilisasi oleh sperma.

* Fertilisasi in vitro dengan preservasi beku embrio ialah pembekuan setelah dilakukan penyatuan sel telur dengan sperma di luar. Sel telur didapat melalui melalui stimulasi ovarium. Hasil persatuan sperma dan telur dibekukan untuk keperluan mendatang.

* Preservasi beku sperma ialah pengambilan sperma secara langsung dari testis atau masturbasi. Kemudian, ejakulat sperma dicuci dan pembekuan sperma dilakukan dalam tabung.

* Preservasi beku jaringan testis ialah prosedur yang ditawarkan pada pasien yang belum menghasilkan sperma. Prosedur ini diharapkan dapat mentransplantasi sel punca spermatogonium kembali ke dalam testis pasien sehingga maturase spermatozoa terjadi di kultur organ testis yang diimplantasi.

* Penanganan sebelum tatalaksana kanker dengan agonis GNRH ialah pemberian agonis GnRH dengan tujuan memperbaiki preservasi ovarium dan menjaga kesuburan.

**Q5 Pengetahuan tentang pilihan-pilihan preservasi kesuburan**

Bagaimana Anda menggambarkan tingkat pengetahuan Anda tentang pilihan-pilihan preservasi kesuburan berikut ini?

| **Mohon centang satu boks untuk masing-masing baris** | **Tidak tahu sama sekali** | **Tahu sedikit** | **Tahu** | **Sangat tahu** |
| --- | --- | --- | --- | --- |
| Preservasi beku jaringan ovarium (*ovarian tissue cryopreservation*) |  |  |  |  |
| Preservasi beku oosit (*oocyte cryopreservation*) |  |  |  |  |
| Fertilisasi in vitro dengan preservasi beku embrio (*in vitro fertilization with embryo cryopreservation*) |  |  |  |  |
| Preservasi beku sperma (*sperm cryopreservation*) |  |  |  |  |
| Preservasi beku jaringan testis (*testicular tissue cryopreservation*) |  |  |  |  |
| Penanganan sebelum tatalaksana kanker (*pre-treatment*) dengan agonis GNRH (seperti suntikan depot leuprolide) |  |  |  |  |

**Q6 Kebutuhan informasi tentang pilihan-pilihan preservasi kesuburan**

Apakah Anda merasa membutuhkan pengetahuan lebih tentang pilihan-pilihan preservasi kesuburan?

- Ya
- Tidak

**Q7 Batas atas usia preservasi kesuburan – perempuan**

Pada kelompok usia yang mana menurut pertimbangan Anda saran preservasi kesuburan relevan bagi seorang perempuan?

- 40
- 45
- 50
- 55
- 60
- Tidak ada batasan

**Q8 Batas atas usia preservasi kesuburan – laki-laki**

Pada kelompok usia yang mana menurut pertimbangan Anda saran preservasi kesuburan relevan bagi seorang laki-laki?

- 40
- 45
- 50
- 55
- 60
- Tidak ada batasan

**Q9 Dari mana Anda mengetahui sumber informasi tertulis/ petunjuk penatalaksanaan mengenai preservasi kesuburan?** (boleh lebih dari 1)

**Q10 Pemberian saran preservasi kesuburan - frekuensi**

Seberapa sering Anda melakukan masing-masing dari yang berikut ini pada pasien usia subur?

|  | **Tidak pernah** | **Jarang** | **Biasanya** | **Selalu** |
| --- | --- | --- | --- | --- |
| Saya mengecek kepada pasien seberapa penting kesuburan mereka di masa mendatang bagi mereka |  |  |  |  |
| Ketika saya merencanakan rejimen penanganan pasien, saya mempertimbangkan keinginan mereka akan kesuburan di masa mendatang |  |  |  |  |
| Saya mendiskusikan kemungkinan dampak kondisi pasien dan/atau penanganan terhadap kesuburan mereka di masa mendatang |  |  |  |  |
| Saya memberi pasien-pasien saya informasi tertulis tentang preservasi kesuburan |  |  |  |  |
| Saya berkonsultasi dengan spesialis fertilitas atau ahli endokrin reproduksi tentang masalah kesuburan pada pasien-pasien saya |  |  |  |  |
| Saya merujuk pasien-pasien yang memiliki pertanyaan tentang kesuburan ke spesialis fertilitas atau ahli endokrin reproduksi |  |  |  |  |

TANYAKAN KEPADA SEMUA

**Q11 Apakah Anda mengetahui keberadaan tim preservasi kesuburan di kota Anda?**

- Ya, di mana? ….
- Tidak

**Q12 Rujukan ke spesialis fertilitas**

Sekitar berapa banyak pasien Anda yang **dirujuk** ke spesialis fertilitas dan/atau telah **menjalani penanganan fertilitas** terkait penanganan kankernya dalam **SATU tahun terakhir**?

Mohon centang satu boks pada masing-masing kolom.

| **Jumlah kasus** | **Rujukan** | **Penanganan** |
| --- | --- | --- |
| 0 |  |  |
| 1-5 |  |  |
| 6-10 |  |  |
| >10 |  |  |

**Q13 Konsultasi panduan masalah kesuburan**

Apakah Anda telah membaca panduan nasional/ internasional untuk arahan tentang masalah kesuburan?

Mohon centang semua yang sesuai.

- Panduan rumah sakit setempat
- NICE *Fertility guidelines* CG11 (2004)
- *RCP, RCR and RCOG Guidance – the effects of cancer treatment on reproductive* *functions* (2007) (tentang efek penanganan kanker pada fungsi reproduktif)
- Lainnya, mohon jelaskan: _____________________________
- Saya tidak membaca panduan apapun

**Q14 Jarak ke tempat rujukan terdekat untuk kedokteran reproduksi**

Sekitar berapa jauh dari klinik Anda unit kedokteran reproduksi/spesialis fertilitas terdekat yang dapat Anda jadikan rujukan? Mohon centang jawaban yang paling sesuai.

- Di rumah sakit yang sama
- Di kota yang sama
- Dalam 40 km
- Dalam 80 km
- Dalam 160 km
- Lebih dari 160 km
- Tidak tahu

**Q15 Konsultasi panduan tentang masalah kesuburan**

Pernyataan yang mana yang paling menggambarkan hubungan profesional Anda dengan unit kedokteran reproduksi yang terdekat dengan Anda?

Mohon centang jawaban yang paling sesuai.

- Sangat baik – saya sangat mengenal mereka dan tahu siapa yang dapat saya hubungi untuk merujuk/mendiskusikan pasien
- Baik – saya tidak banyak berhubungan tapi saya tahu siapa yang dapat saya hubungi untuk merujuk/mendiskusikan pasien
- Kurang baik – saya pernah menghubungi mereka di masa lampau dan tidak memperoleh apa yang saya butuhkan
- Saya tidak tahu – saya belum pernah perlu menghubungi mereka sama sekali

**Q16 Sikap terhadap preservasi kesuburan**

Seberapa Anda setuju atau tidak setuju dengan masing-masing pernyataan berikut ini?

| **Mohon centang satu boks untuk masing-masing baris** | **Sangat tidak setuju** | **Tidak setuju** | **Tidak menyetujui maupun menolak** | **Setuju** | **Sangat setuju** |
| --- | --- | --- | --- | --- | --- |
| Preservasi kesuburan adalah prioritas penting bagi saya untuk saya bicarakan dengan pasien kanker yang baru didiagnosis |  |  |  |  |  |
| Menangani kanker primer lebih penting daripada preservasi kesuburan |  |  |  |  |  |
| Tingkat keberhasilan preservasi kesuburan masih kurang baik untuk mempertimbangkan pilihan tersebut |  |  |  |  |  |
| Saya merasa nyaman membicarakan preservasi kesuburan dengan pasien-pasien saya |  |  |  |  |  |
| Saya bersedia memberikan rejimen penanganan kanker dosis minimal agar dapat mempertahankan kesuburan seorang pasien |  |  |  |  |  |

**Q17 Sikap-sikap terhadap preservasi kesuburan**

Sejauh mana Anda merasa bahwa yang berikut ini menjadi faktor penentu pada pasien-pasien mengenai kesuburannya di masa mendatang?

|  | **Ya** | **Tidak** |
| --- | --- | --- |
| Jenis kelamin |  |  |
| Status sosial ekonomi |  |  |
| Tingkat pendidikan yang sudah dicapai |  |  |
| Latar belakang budaya |  |  |

**Q18a Jenis kelamin yang paling perhatian terhadap preservasi kesuburan**

Menurut Anda perempuan atau laki-laki yang lebih perhatian terhadap preservasi kesuburan?

- Laki-laki
- Perempuan
- Keduanya setara

**Q18b Status sosial ekonomi yang paling perhatian terhadap preservasi kesuburan**

Menurut Anda pasien dengan status sosial ekonomi yang lebih rendah atau yang lebih tinggi yang lebih perhatian terhadap preservasi kesuburan?

- Status sosial ekonomi yang lebih tinggi
- Status sosial ekonomi yang lebih rendah
- Keduanya setara

**Q18c Tingkat pendidikan yang paling perhatian terhadap preservasi kesuburan**

Pasien dari tingkat pendidikan yang mana yang menurut pengalaman Anda paling perhatian terhadap preservasi kesuburan di masa mendatang?

- Sekolah Menengah Atas (SMA) atau sederajat
- Universitas (S1) atau sederajat
- Pascasarjana atau sederajat
- Semua tingkatan setara

**Q18d Latar belakang budaya yang paling perhatian terhadap preservasi kesuburan**

Dari pengalaman Anda pasien dari latar belakang budaya yang mana yang paling memikirkan kesuburan di masa mendatang?

**Q19 Latar belakang budaya yang paling perhatian terhadap preservasi kesuburan**

Sejauh mana Anda berpandangan faktor-faktor berikut ini mempengaruhi apakah Anda akan memulai pembicaraan tentang kesuburan dengan seorang pasien?

| **Centang satu boks untuk masing-masing baris** | **Sama sekali tidak** | **Sampai ke taraf tertentu** | **Sangat mempengaruhi** |  |
| --- | --- | --- | --- | --- |
| Rendahnya tingkat keberhasilan pilihan preservasi kesuburan |  |  |  |  |
| Kurangnya pelayanan fertilitas di daerah setempat |  |  |  |  |
| Keterbatasan waktu saya |  |  |  |  |
| Keterbatasan pengetahuan saya tentang pilihan-pilihan preservasi kesuburan |  |  |  |  |
| Beban pada pasien |  |  |  |  |
| Orang lain di praktik saya membicarakan preservasi kesuburan dengan pasien saya |  |  |  |  |
| Pasiennya | | | | |
| … terlalu berat sakitnya untuk menunda penanganan untuk mengupayakan preservasi kesuburan |  |  |  |  |
| … tidak mampu membayar preservasi kesuburan |  |  |  |  |
| … memiliki keganasan yang sensitif terhadap hormon |  |  |  |  |
| … tidak mau membicarakan preservasi kesuburan |  |  |  |  |
| … prognosis buruk |  |  |  |  |
| … tidak berpasangan |  |  |  |  |
| … sudah punya anak |  |  |  |  |

**Q20 Pengaruh lain dalam memulai pembicaraan tentang preservasi kesuburan**

Apakah ada faktor-faktor lain, terlepas dari yang tertera di atas, yang akan mempengaruhi Anda dalam hal memulai atau tidak memulai pembicaraan tentang kesuburan dengan seorang pasien?

Mohon jelaskan secara lengkap

- Tidak ada faktor-faktor lain

**Q21 Komentar-komentar lainnya**

Mohon tambahkan komentar-komentar lainnya yang mungkin Anda punya tentang preservasi kesuburan dan pasien-pasien Anda:

- Saya tidak ada komentar lebih lanjut tentang hal ini
