# Supplementary material for: Developing an Indonesian fertility preservation questionnaire for health care providers treating patients with cancer: A preliminary pilot study
Source: F1000Res. 2019 May 10;7:1890. Originally published 2018 Dec 4. [Version 2] doi: 10.12688/f1000research.15948.2 (PMC6544131; doi:10.12688/f1000research.15948.2)
Supplement: Peer review history

# Peer review: Reviewer response for version 2

**DOI:** 10.5256/f1000research.21101.r48355

**Published:** 2019-05-28

**Authors:** Jane A. Stewart

**Affiliations:** 1 Newcastle Fertility Centre, International Centre for Life, Newcastle upon Tyne Hospitals NHS Foundation Trust, Newcastle upon Tyne, UK

Thank you for addressing my previous comment. I look forward to hearing more from the use of the questionnaire in practice.

I have read this submission. I believe that I have an appropriate level of expertise to confirm that it is of an acceptable scientific standard.

# Peer review: Reviewer response for version 1

**DOI:** 10.5256/f1000research.17418.r45599

**Published:** 2019-04-11

**Authors:** Jane A. Stewart

**Affiliations:** 1 Newcastle Fertility Centre, International Centre for Life, Newcastle upon Tyne Hospitals NHS Foundation Trust, Newcastle upon Tyne, UK

This paper describes a pilot study of a questionnaire to assess the practice of health care providers' (namely oncologists') understanding and attitudes to fertility preservation in a local setting to consider its utility nationally. The authors describe the process of translation and verification of sense of the questionnaire and then its use in the clinical setting with a small group of oncologists. The process proved effective.

 The initial findings are of interest and the authors have raised a number of relevant points which confirm that rolling out nationally may indeed be informative.

 The authors see the trial as successful. They comment on the discussion around cultural questions. They have not discussed the comments around the "difficulty" of the questionnaire which more than one respondent made. Since the paper was designed to explore the utility of the questionnaire rather than the specific answers given by the oncologists it would be useful to see more discussion around those points and in particular whether or not they felt that any modification was needed before wider use in Indonesia. If so what and if not, given those comments, why not?

I have read this submission. I believe that I have an appropriate level of expertise to confirm that it is of an acceptable scientific standard, however I have significant reservations, as outlined above.

# Response

**Published:** 2019-05-06

**Authors:** Achmad Kemal

**Affiliations:** RSUPN Dr. Cipto Mangunkusumo Hospital, Indonesia

Thank you for the review.

We uploaded revision on our manuscript. We hope to hear your review.

# Peer review: Reviewer response for version 1

**DOI:** 10.5256/f1000research.17418.r42438

**Published:** 2019-01-22

**Authors:** Yutaka Osuga

**Affiliations:** 1 Department of Obstetrics and Gynecology, Faculty of Medicine, University of Tokyo, Tokyo, Japan

In this study, the authors aimed to develop a standardized Indonesian questionnaire from already published English questionnaire that was successfully used to survey oncologists’ current knowledge, practice and attitudes in a Western country. The authors translated the questionnaire, and the content was validated through the process of back translation. In addition, the authors used the translated questionnaire to interview ten sub-specialists in Indonesia. The questionnaire worked well for that purpose.

 In summary, the translated questionnaire will be useful for future studies in the field of oncofertility in Indonesia and may be used for comparing the quality of health care providers’ knowledge, attitude, and practice regarding fertility preservation in patients with cancer in different countries.

I have read this submission. I believe that I have an appropriate level of expertise to confirm that it is of an acceptable scientific standard.
